# Progesterone and Androstenedione Are Important Follicular Fluid Factors Regulating Porcine Oocyte Maturation Quality

**DOI:** 10.3390/ani13111811

**Published:** 2023-05-30

**Authors:** Huaxing Zhao, Xiaohua He, Xianjun Zhang, Junsong Shi, Rong Zhou, Ranbiao Mai, Qiaoyun Su, Gengyuan Cai, Sixiu Huang, Zheng Xu, Zhenfang Wu, Zicong Li

**Affiliations:** 1 National Engineering Research Center for Breeding Swine Industry, South China Agricultural University, Guangzhou 510642, China; zhaohuaxing@stu.scau.edu.cn (H.Z.);; 2 State Key Laboratory of Livestock and Poultry Breeding, South China Agricultural University, Guangzhou 510642, China; 3 Guangdong Provincial Key Laboratory of Agro-Animal Genomics and Molecular Breeding, South China Agricultural University, Guangzhou 510642, China; 4 Department of Animal Genetics, Breeding and Reproduction, College of Animal Science, Guangzhou 510642, China; 5 Guangdong Wens Breeding Swine Technology Co., Ltd., Yunfu 527439, China; 6 Guangdong Laboratory for Lingnan Modern Agriculture, Guangzhou 510642, China

**Keywords:** pig, oocyte maturation, follicular fluid, maturation medium, metabolomics

## Abstract

**Simple Summary:**

The quality of in vitro matured oocytes is usually lower than that of in vivo matured oocytes, possibly due to the absence of some important signal regulators in vitro. In this study, we found that progesterone and androstenedione were upregulated in FF during in vivo pig oocyte maturation. The supplementation of progesterone or androstenedione during in vitro maturation significantly improved the pig oocyte maturation rate or subsequent embryo developmental competence.

**Abstract:**

Oocytes matured in vitro are useful for assisted human and farm animal reproduction. However, the quality of in vitro matured oocytes is usually lower than that of in vivo matured oocytes, possibly due to the absence of some important signal regulators in vitro. In this study, untargeted metabolomics was used to detect the changes in the metabolites in the follicular fluid (FF) during in vivo pig oocyte maturation and in the culture medium during in vitro maturation. Our results showed that the total metabolite changing profile of the in vivo FF was different from that of the in vitro maturation medium, but the levels of 23 differentially expressed metabolites (DEMs) changed by following the same trend during both in vivo and in vitro pig oocyte maturation. These 23 metabolites may be important regulators of porcine oocyte maturation. We found that progesterone and androstenedione, two factors in the ovarian steroidogenesis pathway enriched from the DEMs, were upregulated in the FF during in vivo pig oocyte maturation. The levels of these two factors were 31 and 20 fold, respectively, and they were higher in the FF than in the culture medium at the oocyte mature stage. The supplementation of progesterone and androstenedione during in vitro maturation significantly improved the pig oocyte maturation rate and subsequent embryo developmental competence. Our finding suggests that a metabolic abnormality during in vitro pig oocyte maturation affects the quality of the matured oocytes. This study identified some important metabolites that regulate oocyte maturation and their developmental potential, which will be helpful to improve assisted animal and human reproduction.

## 1. Introduction

In vitro matured oocytes play an important role in farm animal reproduction [1], human-assisted reproduction [2], and biological science [3] because they can be used to produce in vitro fertilization, parthenogenetic activation, and cloned embryos. However, the embryos generated with in vitro matured oocytes usually exhibit a lower developmental potential than those derived from in vivo matured oocytes [4,5,6,7]. This suggests that the quality of in vitro matured oocytes is inferior compared to that of their in vivo matured counterparts.

The quality difference between in vitro and in vivo matured oocytes is mainly caused by the differences in the maturation environment between these two types of oocytes. Although the in vitro oocyte maturation condition has been improved in the past decades, it still cannot fully mimic the in vivo oocyte maturation condition. For example, the in vitro oocyte maturation medium lacks Stromal cell-derived factor 1 (SDF1) [8], growth differentiation factor 9 (GDF9) [9], bone morphogenetic protein 15 (BMP15) [9], neurotrophic factors [10], and melatonin [11], which all are present in in vivo follicular fluid (FF) and are critical for oocyte maturation quality.

FF is an important microenvironment for in vivo matured oocytes. FF contains various regulatory molecules such as RNAs, proteins, metabolites, and hormones, which are transported from the blood or are secreted by follicular cells [12,13]. The level of many molecules in the FF is changed dynamically during in vivo oocyte maturation [14,15,16,17]. The abundance of some factors in the FF has been shown to be correlated with the quality or developmental ability of mature oocytes [18,19,20,21].

In this study, we analyzed the FF metabolic profile of porcine in vivo mature and immature follicles and the culture medium metabolic profile of porcine in vitro mature and immature oocytes. After a metabolomics analysis, two metabolites enriched in the ovarian steroidogenesis pathway, including progesterone and androstenedione, were selected to further investigate their roles in pig oocyte maturation quality. We showed that the addition of progesterone and androstenedione into a pig oocyte in vitro maturation medium has positive effects on improving the oocyte maturation rate and subsequent embryo development.

## 2. Materials and Methods

### 2.1. Chemicals

Unless otherwise mentioned, all chemicals used in the experiment were procured from Sigma-Aldrich (Saint Louis, MO, USA).

### 2.2. Collection of In Vivo Follicular Fluid and In Vitro Oocyte Culture Medium

The collection of the in vivo follicular fluid and in vitro oocyte culture medium were performed as previously described [5,8]. Briefly, Duroc sows (about 14 months old) were treated with altrenogest (Yofoto, Ningbo, China) at 20 mg per sow per day for 18 days, and then they were injected with 1000 IU of PMSG (Yofoto, Ningbo, China). Forty hours after the PMSG injection, estrous sows were anesthetized and their ovaries were exposed via surgery. The pig mature follicular fluid (pMFF) was collected from preovulary follicles (diameter > 15 mm) in the ovaries with an obvious ovulation point. The pig immature follicular fluid (pIFF) was collected from immature follicles (3 mm < diameter < 5 mm) of nonestrous 14-month-old Duroc sows via surgery. Eight pMFF samples and nine pIFF samples were collected from 8 estrous and 9 anestrous sows, respectively. Eight pig oocyte mature medium (pMM) samples were collected from the IVM medium of porcine COCs cultured for 44 h, centrifuged, and stored in liquid nitrogen; eight immature pig oocyte medium (pIM) samples were collected from the fresh IVM medium of porcine COCs, centrifuged, and stored in liquid nitrogen.

### 2.3. Ultra-High-Performance Liquid Chromatography-Mass Spectrometry (UHPLC-MS)/MS Analysis

A total of 100 μL of the follicular fluid or mature medium samples was taken out from the liquid nitrogen and thawed slowly at 4 °C. In total, 400 μL of prechilled (−20 °C) 80% methanol was added. The mixed liquor was vortexed, incubated at −20 °C for 60 min, and then centrifuged at 14,000× *g* at 4 °C for 20 min. The supernatants were subsequently transferred to a fresh centrifuge tube. All the samples were mixed in equal volume as quality control samples to assess the stability of the samples over the entire experiment period before testing. The supernatants of the follicular fluid or medium were analyzed via LC-MS/MS. An LC-MS/MS analysis was completed by Beijing Novogene Technology Co., Ltd. (Beijing, China) using high performance liquid chromatograph (Vanquish UHPLC, Thermo Fisher, Waltham, MA, USA) and mass spectrometer (QE-HF-X, Thermo Fisher, Waltham, MA, USA).

The peak alignment, peak selection, and quantification of each metabolite from the raw data file generated by UHPLC-MS/MS were obtained by using compound Discoverer 3.0 (CD 3.0, Thermo Fisher, Waltham, MA, USA). The molecular formula was predicted based on additive ions, molecular ion peaks, and fragment ions by using normalized data. For the metabolites that responded more strongly to positive or negative ions, we chose the mode with a stronger response as the detection result of this metabolite. Peak matching was performed by using mzCloud (“https://www.mzcloud.org/” accessed on 23 February 2019) and ChemSpider (“http://www.chemspider.com/” accessed on 23 February 2019) databases to obtain accurate qualitative and relative quantitative results. The data were imported into EZinfo software (version 2.0; Umetrics AB, Umeå, Sweden) for a principal component analysis (PCA). The first principal component of the PLS-DA model was used to obtain the variable importance in projection (VIP) value. The *p* values of the metabolites in the two groups were analyzed by using Student’s *t*-tests. Finally, the differentially expressed metabolites were identified by using the following criteria: *p* value < 0.05, VIP value >1 and fold change ≥2. 

The Kyoto encyclopedia of genes and genomes enrichment (KEGG) analysis was performed by using hypergeometric test to identify pathways that were significantly enriched in the differentially expressed metabolites compared with the background of the total identified metabolites.

### 2.4. Preparation of In Vitro Matured Oocytes

The collection of COCs was performed as previously described [8]. Briefly, pig COCs were obtained from the ovaries via aspiration by using an 18-gauge needle attached to a 10 mL disposable syringe. The COCs that met the criteria of having at least three layers of compact cumulus cells and intact cytoplasm were selected for IVM. Approximately 50~60 COCs were transferred to each well of a four-well Nunc dish into 500 μL of fresh IVM medium. The IVM medium was prepared by supplementing Medium-199 with 10 ng/mL epidermal growth factor (EGF), 40 ng/mL fibroblast growth factor 2 (FGF2) (PeproTech, Suzhou, China), 20 ng/mL leukemia inhibitory factor (LIF) (PeproTech, Suzhou, China), 20 ng/mL insulin-like growth factor (IGF) (PeproTech, Suzhou, China), 5 μg/mL inositol, an ITS-liquid medium supplement (1×), 10 IU/mL pregnant mare serum gonadotropin (PMSG) (Yofoto, Ningbo, China), 10 IU/mL luteinizing-hormone-releasing hormone A3 (Yofoto, Ningbo, China), and 0.6 mM cysteine, and it was cultured at 38.5 °C with 5% CO_2_ for 44 h in a humidified atmosphere. The COCs were incubated in Dulbecco’s phosphate buffered saline (DPBS) containing 0.1% polyvinyl alcohol (PVA) and 1 mg/mL hyaluronidase for 5 min, followed by gentle pipetting approximately 200 times to remove the surrounding cumulus cells. The oocytes were examined by using a stereomicroscope and were considered matured when the first polar body was visible in the perivitelline space. These oocytes were utilized for the preparation of cloning, in vitro fertilization (IVF), and parthenogenetic activation (PA) embryos. 

### 2.5. Production of Embryos

The production of PA, IVF, and cloned embryos were performed as previously described [8]. The production of PA embryos occurred as follows: the matured pig oocytes were electrically activated using two direct current pulses of 85 V/mm for 100 μs in 0.28 mol/L mannitol supplemented with 0.1 mM MgSO_4_ and 0.1% PVA. The production of IVF embryos occurred as follows: the capacitated sperm (final concentration: 1 × 10^5^ sperm/mL) were added to the well containing the matured oocytes in a humidified incubator for six hours at 38.5 °C under 5% CO_2_. The production of cloned embryos occurred as follows: the nuclei of the matured oocytes were stained with 1 g/mL Hoechst 33,342, removed using a microinjection needle, and then a round donor cell that was slightly spiculated was selected and injected into the perivitelline space of the enucleated oocytes. Finally, the reconstructed oocytes were activated in 0.28 mol/L mannitol supplemented with 0.1 mM MgSO_4_ and 0.1% PVA by using two direct current pulses of 150 V/mm for 50 μs.

All the embryos were washed three times in a porcine zygote medium-3 (PZM-3) medium and were then cultured in a PZM-3 medium at 38.5 °C under 5% CO_2_ and saturated humidity, and the cleavage and blastocyst rates were assessed two and six days after embryo production.

### 2.6. Experimental Design

Experiment 1: Porcine COCs were cultured in the IVM medium supplemented with 100 μM progesterone. After a 44 h culture, the number of oocytes that excreted the first polar body was calculated under a stereoscope. The cleavage and blastocyst rates were assessed two and six days after PA and SCNT embryo production.

Experiment 2: Porcine COCs were cultured in the IVM medium supplemented with 0 ng/mL, 5 ng/mL, 75 ng/mL, 125 ng/mL, and 250 ng/mL androstenedione. After the 44 h culture, the number of oocytes that excreted the first polar body was calculated under a stereoscope. The cleavage and blastocyst rates were assessed two and six days after embryo production.

### 2.7. Statistical Analysis

The data on the oocyte maturation rate and embryo development were analyzed with chi-square test. The data on the progesterone, androstenedione, and testosterone in the pIFF, pMFF, pIM, and pMM were compared between different groups by using an ANOVA with LSD by using IBM SPSS Statistics 27 software. Only *p* < 0.05 was considered statistically significant.

## 3. Results

### 3.1. Untargeted Metabolomics Analysis of Porcine In Vivo Follicular Fluid

**Figure 1:**
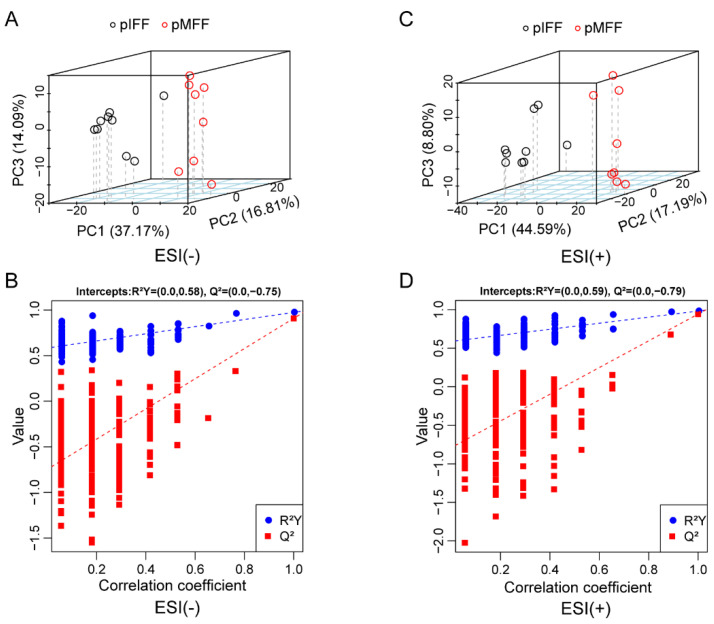
PCA and permutation test plots for the pMFF and pIFF. PCA plot (**A**) and permutation test plot (**B**) for the pMFF and pIFF in the negative ion model. PCA plot (**C**) and permutation test plot (**D**) for pMFF and pIFF in the positive ion model.

The principal component analysis (PCA) indicated significant differences in the metabolic profile between the pMFF and pIFF samples regarding their negative (Figure 1A) and positive (Figure 1C) ion models. In addition, permutation test showed that R^2^Y = 0.58, |Q^2^|= 0.75 in the negative ion model (Figure 1B) and R^2^Y = 0.59, |Q^2^| = 0.79 in the positive ion model (Figure 1D), which suggests that the ion models established in this study were reliable. 

**Figure 2:**
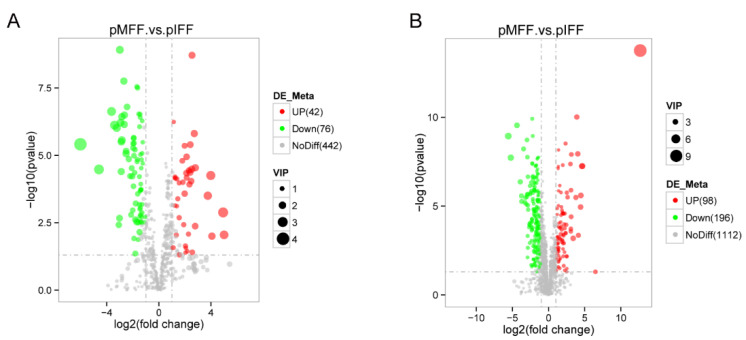
Volcano plot of differentially expressed metabolites between pMFF and pIFF groups in the negative ion model (**A**) or positive ion model (**B**).

Differentially expressed metabolites (DEMs) in the FF were screened based on the variable importance in projection (VIP) values > 1, *p*-value < 0.05 and |log2(fold change)| > 1. In total, 118 metabolites (42 upregulated and 76 downregulated) were identified in the negative ion model (Figure 2A) and 294 metabolites (98 upregulated and 196 downregulated) were identified in the positive ion model (Figure 2B).

### 3.2. Untargeted Metabolomics Analysis of Porcine Oocyte In Vitro Culture Medium

**Figure 3:**
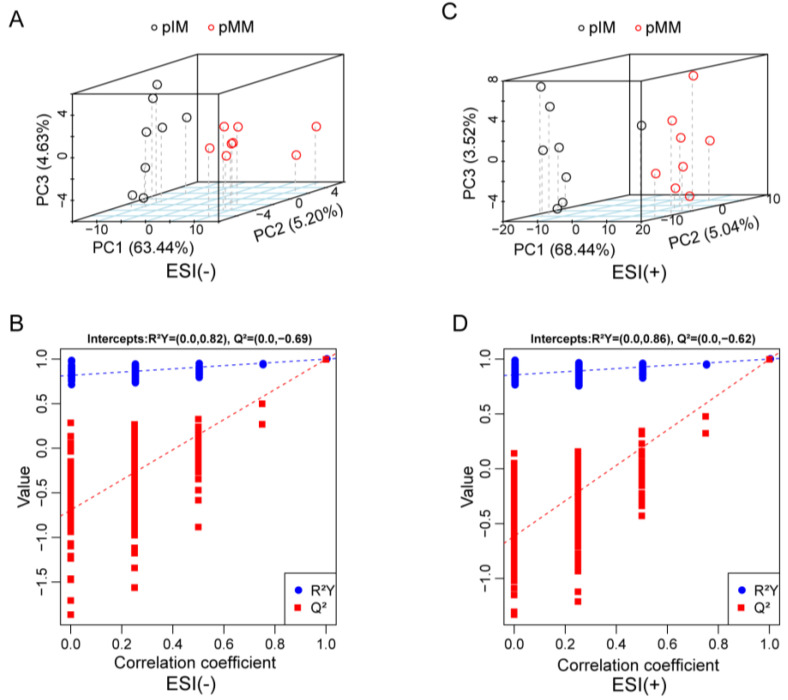
PCA and permutation test plots for the pMM and pIM. PCA plot (**A**) and permutation test plot (**B**) for the pMM and pIM in the negative ion model. PCA plot (**C**) and permutation test plot (**D**) for pMM and pIM in the positive ion model.

The PCA indicated significant differences in the metabolic profile between the pMM and pIM samples regarding their negative (Figure 3A) and positive (Figure 3C) ion models. A permutation test showed that R^2^Y = 0.82, |Q^2^| = 0.69 in the negative ion model (Figure 3B) and R^2^Y = 0.86, |Q^2^|= 0.62 in the positive ion model (Figure 3D), which suggests that the models were reliable for subsequent analysis. 

**Figure 4:**
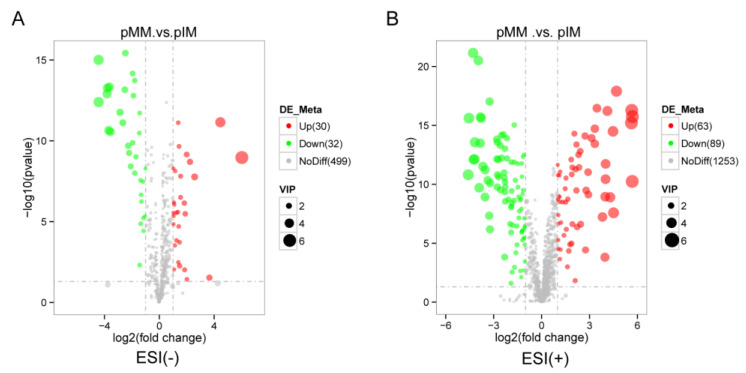
Volcano plot of differentially expressed metabolites (DEMs) between pMM and pIM groups in the negative ion model (**A**) or positive ion model (**B**).

In the porcine oocyte in vitro culture medium, 62 DEMs (31 upregulated and 32 downregulated) were identified in the negative ion model (Figure 4A) and 152 DEMs (63 upregulated and 89 downregulated) were identified in the positive ion model (Figure 4B).

### 3.3. Metabolites with a Same Change Trend during In Vivo and In Vitro Pig Oocyte Maturation

**Figure 5:**
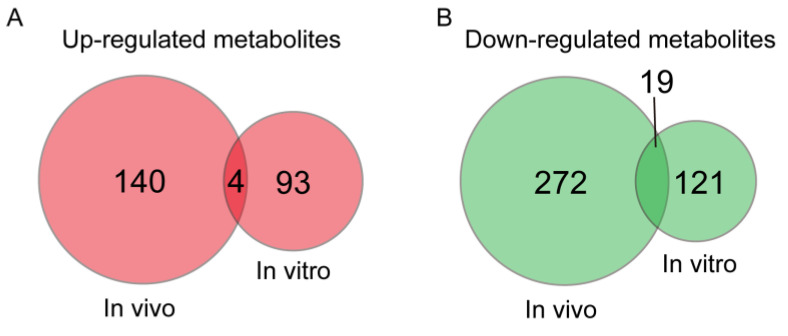
The Venn diagram of metabolites upregulated (**A**) and downregulated (**B**) in in vivo follicular fluid and in vitro culture medium during oocyte maturation. The 23 metabolites with a same level change trend are shown in Table 1.

The metabolites changed and followed the same trend during both in vivo and in vitro pig oocyte maturation, and this probably plays an important role in regulating the oocyte maturation quality. To identify these potential critical metabolites, a Venn analysis was conducted. The results showed that 4 and 19 metabolites were upregulated and downregulated, respectively, during both in vivo and in vitro oocyte maturation (Figure 5). These 23 possible important metabolites are shown in Table 1.

### 3.4. Untargeted Metabolomics Analysis of Porcine In Vivo Follicular Fluid

To investigate the functions of the identified DEMs, a Kyoto encyclopedia of genes and genomes (KEGG) enrichment analysis was performed. The DEMs in the follicular fluid during oocyte in vivo maturation were enriched in the metabolic pathways during the degradation of compounds, pyrimidine metabolism, and microbial metabolism in diverse environments (negative ion mode; Figure 5A). They were also enriched during ovarian steroidogenesis and pyrimidine metabolism; during alanine, aspartate, and glutamate metabolism; during the biosynthesis of unsaturated fatty acids; when in the prolactin signaling pathway; during endocrine resistance; during steroid degradation; and during galactose metabolism (positive ion model; Figure 5B).

The DEMs in the culture medium during oocyte in vitro maturation were enriched in two metabolic pathways, including “stilbenoid, diaryheptanoid and gingerol biosynthesis” and “glucocorticoid and mineralocorticoid receptor agonists/antagonists” (negative ion model; Figure 5C). 

### 3.5. Untargeted Metabolomics Analysis of Porcine In Vivo Follicular Fluid

**Figure 6:**
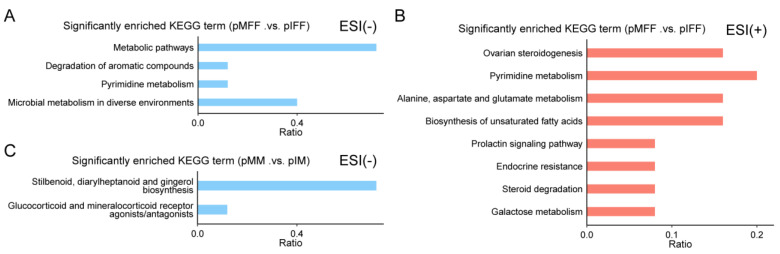
KEGG functional enrichment analysis of DEMs. KEGG functional enrichment analysis of DEMs for pMFF and pIFF in the negative ion model (**A**) and positive ion model (**B**). KEGG functional enrichment analysis of DEMs for pMM and pIM in the negative ion model (**C**).

**Figure 7:**
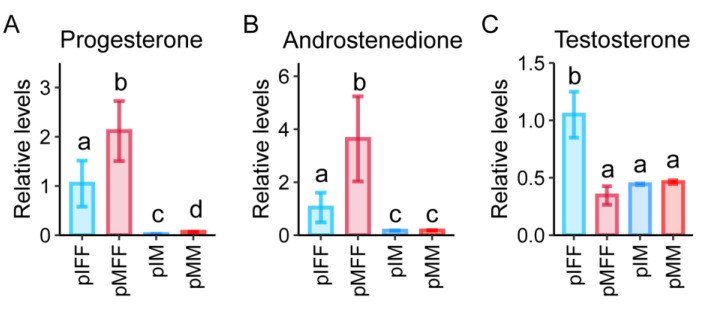
The levels of DEMs enriched in the KEGG pathway of ovarian steroidogenesis. Values labeled with different letters differ at *p* < 0.05.

Among the four DEMs that were upregulated during both in vivo and in vitro pig oocyte maturation (Table 1), progesterone attracted our interest because it was also enriched in the KEGG pathway of ovarian steroidogenesis (Figure 6). This suggests that the ovarian steroidogenesis pathway may participate in regulating porcine oocyte maturation. The levels of the three DEMs enriched in the ovarian steroidogenesis pathway, including progesterone, androstenedione, and testosterone, are shown in Figure 7. Progesterone and androstenedione were significantly upregulated while testosterone was significantly downregulated in the FF during in vivo oocyte maturation (Figure 7). Although progesterone was also significantly upregulated in the culture medium during in vitro oocyte maturation, its level was about 31-fold higher in the pMFF than in the pMM (Figure 7A). The level of androstenedione in the pMFF was approximately 20-fold higher than that in the pMM (Figure 7B). This implies that the levels of progesterone and androstenedione in the pig oocyte in vitro culture medium were insufficient compared to those in the FF.

Progesterone and androstenedione were added into the in vitro culture medium so we could study their effects on the pig oocyte maturation quality. As shown in Table 2, the supplementation of 100 μM of progesterone during IVM significantly increased the rate of oocytes extruding the first polar body (71.63% vs. 58.70%, *p* < 0.05). However, the supplementation of progesterone during IVM did not affect the subsequent development of PA embryos (Table 3) and cloned embryos (Table 4).

The addition of 5, 25, 125, and 250 ng/mL androstenedione into the IVM medium did not affect the porcine oocyte maturation rate (Table 5). Supplementation with 125 ng/mL androstenedione significantly improved the blastocyst formation rate of PA embryos (50.11% vs. 34.72%, *p* < 0.05; Table 6). Supplementation with 125 ng/mL androstenedione during pig oocyte IVM significantly enhanced the cleavage rate (57.63% vs. 42.84%, *p* < 0.05) but had no significant effect on the blastocyst formation rate (8.65% vs. 9.86%, *p* > 0.05) of IVF embryos (Table 7).

## 4. Discussion

We demonstrated in this study that porcine ovarian follicular fluid contains elements that are beneficial for the oocyte maturation rate and developmental competence. Similar results were found in our previous studies [5,8]. We also showed that a metabolomics analysis is a powerful tool that can be used to find the molecules regulating the oocyte maturation quality in FF or in a maturation medium.

Our data indicated that the levels of 412 metabolites were changed significantly in the FF during pig oocyte in vivo maturation. Nevertheless, during in vitro pig oocyte maturation, only 214 DEMs were detected in the culture medium. These results suggest that more metabolites in the FF are changed dynamically during in vivo oocyte maturation than in the culture medium during in vitro oocyte maturation. Four DEMs that were upregulated and nineteen DEMs that were downregulated during both in vivo and in vitro pig oocyte maturation was identified in the present study. These 23 metabolites may play important roles in pig oocyte maturation. Among them, at least six metabolites, including vitamin C [22,23], progesterone [24,25], 8-hydroxy-deoxyguanosine [26,27], putrescine [28], N-Acetyl-L-cysteine [28,29], and hypotaurine [30,31] have been reported to regulate oocyte maturation rate or quality. 

In the positive ion model, the KEGG pathways enriched from the DEMs in the FF were different from those enriched from the DEMs in the maturation medium. In addition, the DEMs in the FF detected by the negative ion model were enriched in eight KEGG pathways, yet no KEGG pathway was enriched from the DEMs in the maturation medium identified by the same ion model. These results suggest that the global metabolic profile between the in vivo ovarian FF and in vitro oocyte maturation medium is different. This difference probably mainly resulted from the difference in the maturation environment between the in vivo and the in vitro matured oocytes. During in vivo maturation, granulosa cells, cumulus cells, oocytes, and other somatic cells secrete various metabolites into the FF to modulate oocyte maturation [12,32,33]. However, during in vitro maturation, granulosa cells and other follicular somatic cells are absent, which results in an abnormal metabolite profile in the culture medium and decreased oocyte maturation quality.

Our results showed that during in vivo pig oocyte maturation, the DEMs in follicular fluid were enriched in a metabolic pathway called alanine, aspartate, and glutamine metabolism. A previous study also found that the alanine, aspartate, and glutamine metabolism pathway is disturbed and the glutamine level is decreased in follicular fluid from low reproductive sows compared to that from normal reproductive sows [34]. Glutamine is a precursor of glutathione [35], which is significantly reduced in follicular fluid during human in vivo oocyte maturation [36] and during in vivo pig oocyte maturation, as shown in the present study. Gamma-glutamylvaline is regulated by glutathione and its precursor glutamine, and the upregulation of gamma-glutamylvaline in aged follicular fluid is an important factor that impairs the oocyte quality [37]. These results suggest that the alanine, aspartate, and glutamine metabolism pathway is involved in regulating human and porcine oocyte maturation. The progesterone concentration in porcine follicular fluid is positively correlated with the developmental potential of oocytes [38]. The addition of progesterone into the porcine oocyte IVM medium enhances subsequent embryo development, while the supplementation of RU486, a specific progesterone inhibitor, during IVM prevents porcine oocyte maturation and reduces the subsequent embryo developmental rate [24]. In addition, the supplementation of progesterone during IVM could rescue the negative effects of estradiol-17 beta on pig oocyte maturation [39]. All these results indicate that the progesterone level in the FF is a biological indicator of pig oocyte maturation quality. We found that although the progesterone abundance was increased in the FF and in IVM medium during in vivo and in vitro pig oocyte maturation, the concentration of progesterone in the in vivo FF was much higher than that in the in vitro culture medium. This implies that in the pig oocyte IVM medium, progesterone is insufficient. In this study, the supplementation with progesterone during IVM significantly improved the pig oocyte maturation rate, which confirms the positive effects of progesterone on pig oocyte maturation.

We showed that androstenedione, another element in the ovarian steroidogenesis pathway, is significantly upregulated in FF during pig oocyte in vivo maturation but not in the IVM medium during in vitro maturation. Previous studies have demonstrated that one’s androstenedione level is associated with follicular development and oocyte capacitation [38,40,41]. In this study, we found that supplementation with 125 ng/mL androstenedione during pig oocyte IVM effectively promoted subsequent embryo development. 

## 5. Conclusions

In conclusion, the changing patterns of the metabolites in the FF during pig oocyte in vivo maturation was different from that in the culture medium during in vitro maturation. The ovarian steroidogenesis pathway plays an important role in modulating pig oocyte maturation as the supplementation of two factors in this pathway, progesterone and androstenedione, during IVM increased the pig oocyte maturation rate and subsequent embryo developmental ability. This study helped identify the key molecules that regulate the oocyte maturation quality, which is beneficial for animal production, human health, and biological science.

## Figures and Tables

**Table 1:** Potential differential metabolites associated with porcine oocyte maturation events.

Number	Metabolites	log2FC (pMFF vs. pIFF)	log2FC (pMM vs. pIM)	Up/ Down	Ion Modern
1	(2R)-2-Hydroxy-3-(phosphonooxy) propyl (8Z,11Z,14Z)-8,11,14-Icosatrienoate	1.84	1.16	Up	Negative
2	Prostaglandin A1 ethyl ester	1.91	1.03	Up	Negative
3	Progesterone	1.36	1.51	Up	Positive
4	Oxprenolol	2.23	1.14	Up	Positive
5	Maleic acid	−1.80	−1.16	Down	Negative
6	Vitamin C	−1.91	−1.37	Down	Negative
7	5-(4-acetoxybut-1-ynyl)-2,2′-Bithiophene	−6.02	−1.18	Down	Negative
8	Glycerol 3-phosphate	−2.69	−1.30	Down	Negative
9	Sinapyl alcohol	−2.12	−2.50	Down	Positive
10	8-hydroxy-deoxyguanosine	−2.931	−1.05	Down	Positive
11	Putrescine	−1.18	−1.72	Down	Positive
12	S-3-oxodecanoyl cysteamine	−1.57	−1.48	Down	Positive
13	N-Acetyl-L-cysteine	−2.69	−1.16	Down	Positive
14	N-Acetylcadaverine	−1.25	−2.29	Down	Positive
15	(2S)-2-Amino-5-({(2R)-3-{[(2R)-2-amino-2-Carboxyethyl]disulfanyl}-1-[(carboxymethyl)amino]-1-oxo-2-propanyl}amino)-5-Oxopentanoic acid	−1.65	−2.16	Down	Positive
16	2E-Crotamiton	−2.86	−4.54	Down	Positive
17	Hypotaurine	−1.95	−1.30	Down	Positive
18	Tetraacetylethylenediamine	−1.50	−1.05	Down	Positive
19	3,4-dehydrothiomorpholine-3-Carboxylic acid	−1.87	−1.05	Down	Positive
20	Guanine	−3.26	−4.24	Down	Positive
21	3-Methylhistamine	−2.02	−1.73	Down	Positive
22	3,3-Dimethyl-1,2-dithiolane	−3.45	−4.59	Down	Positive
23	Phosphonoacetaldehyde	−2.31	−1.11	Down	Positive

**Table 2:** Effects of supplementation of progesterone during IVM on pig oocyte maturation rate.

Groups	No. of Cultured Oocytes	No. of Matured Oocytes	Maturation Rate (%)
Control	644	378	58.70 ^a^
Progesterone	678	414	71.63 ^b^

Oocytes cultured with the IVM medium for 44 h and showing the first polar body in their perivitelline space were considered mature oocytes. Values in the same column labeled with different superscripts differ at *p* < 0.05.

**Table 3:** Effects of supplementation of progesterone during porcine oocyte IVM on subsequent development of PA embryos.

Groups	No. of Cultured Embryos	No. of Cleaved Embryos (%)	No. of Blastocytes (%)
Control	100	77 (77.00)	33 (33.00)
Progesterone	100	74 (74.00)	33 (33.00)

**Table 4:** Effects of supplementation of progesterone during porcine oocyte IVM on subsequent development of cloned embryos.

Groups	No. of Cultured Embryos	No. of Cleaved Embryos (%)	No. of Blastocytes (%)
Control	231	171 (74.03)	33 (14.29)
Progesterone	214	160 (74.77)	35 (16.36)

**Table 5:** Effects of supplementation of androstenedione during IVM on pig oocyte maturation rate.

Concentration (ng/mL)	No. of Cultured Oocytes	No. of Matured Oocytes	Maturation Rate (%)
0	627	440	71.08
5	640	463	72.69
75	576	412	72.71
125	635	455	71.72
250	622	340	67.83

Oocytes cultured with the IVM medium for 44 h and showing the first polar body in their perivitelline space were considered mature oocytes.

**Table 6:** Effects of supplementation of androstenedione during porcine oocyte IVM on subsequent development of PA embryos.

Concentration (ng/mL)	No. of Cultured Embryos	No. of Cleaved Embryos (%)	No. of Blastocytes (%)
0	110	103 (93.46)	38 (34.72 ^a^)
5	104	100 (95.98)	48 (46.40 ^ab^)
75	105	100 (94.79)	49 (46.67 ^ab^)
125	117	103 (88.39)	58 (50.11 ^b^)
250	108	100 (93.50)	40 (36.70 ^a^)

Values in the same column labeled with different superscripts differ at *p* < 0.05.

**Table 7:** Effects of supplementation of androstenedione during porcine oocyte IVM on subsequent development of IVF embryos.

Concentration (ng/mL)	No. of Cultured Embryos	No. of Cleaved Embryos (%)	No. of Blastocytes (%)
0	112	48 (42.84 ^a^)	11 (9.86)
125	139	80 (57.63 ^b^)	12 (8.65)

Values in the same column labeled with different superscripts differ at *p* < 0.05.

## Data Availability

The corresponding author can provide access to the datasets upon request.

## Abbreviations

COCs, cumulus cell–oocyte complexes; DEMs, differentially expressed metabolites; ESI, electrospray ionization; FF, follicular fluid; KEGG, Kyoto encyclopedia of genes and genomes enrichment; IVF, in vitro fertilization; IVM, in vitro maturation; PA, parthenogenetic activation; pIFF, pig immature follicular fluid; pIM, pig oocyte immature medium; pMFF, pig mature follicular fluid; pMM, pig oocyte mature medium; PCA, principal component analysis; PVA, polyvinyl acetate; VIP, variable importance in projection.
